# Fe-Doped g-C_3_N_4_/Bi_2_MoO_6_ Heterostructured Composition with Improved Visible Photocatalytic Activity for Rhodamine B Degradation

**DOI:** 10.3390/molecules29112631

**Published:** 2024-06-03

**Authors:** Chien-Yie Tsay, Ching-Yu Chung, Chi-Jung Chang, Yu-Cheng Chang, Chin-Yi Chen, Shu-Yii Wu

**Affiliations:** 1 Department of Materials Science and Engineering, Feng Chia University, Taichung 40724, Taiwan; icedreamer313@mail.fcu.edu.tw (C.-Y.C.); yuchchang@fcu.edu.tw (Y.-C.C.); chencyi@fcu.edu.tw (C.-Y.C.); 2 Department of Chemical Engineering, Feng Chia University, Taichung 40724, Taiwan; changcj@fcu.edu.tw (C.-J.C.); sywu@fcu.edu.tw (S.-Y.W.)

**Keywords:** Fe-doped g-C_3_N_4_, Bi_2_MoO_6_, heterostructured composite, visible light photocatalyst, organic pollutant, cycling test

## Abstract

The binary heterostructured semiconducting visible light photocatalyst of the iron-doped graphitic carbon nitride/bismuth molybdate (Fe-g-C_3_N_4_/Bi_2_MoO_6_) composite was prepared by coupling with Fe-doped g-C_3_N_4_ and Bi_2_MoO_6_ particles. In the present study, a comparison of structural characteristics, optical properties, and photocatalytic degradation efficiency and activity between Fe-doped g-C_3_N_4_ particles, Bi_2_MoO_6_ particles, and Fe-g-C_3_N_4_/Bi_2_MoO_6_ composite was investigated. The results of X-ray diffraction (XRD) examination indicate that the hydrothermal Bi_2_MoO_6_ particles have a single orthorhombic phase and Fourier transform infrared (FTIR) spectroscopy analysis confirms the formation of Fe-doped g-C_3_N_4_. The optical bandgaps of the Fe-doped g-C_3_N_4_ and Bi_2_MoO_6_ particles are 2.74 and 2.73 eV, respectively, as estimated from the Taut plots obtained from UV-Vis diffuse reflectance spectroscopy (DRS) spectra. This characteristic indicates that the two semiconductor materials are suitable for absorbing visible light. The transmission electron microscopy (TEM) micrograph reveals the formation of the heterojunction Fe-g-C_3_N_4_/Bi_2_MoO_6_ composite. The results of photocatalytic degradation revealed that the developed Fe-g-C_3_N_4_/Bi_2_MoO_6_ composite photocatalyst exhibited significantly better photodegradation performance than the other two single semiconductor photocatalysts. This property can be attributed to the heterostructured nanostructure, which could effectively prevent the recombination of photogenerated carriers (electron–hole pairs) and enhance photocatalytic activity. Furthermore, cycling test showed that the Fe-g-C_3_N_4_/Bi_2_MoO_6_ heterostructured photocatalyst exhibited good reproducibility and stability for organic dye photodegradation.

## 1. Introduction

Nanosized semiconducting photocatalysts exhibit environmental harmlessness, are stable in photochemical reactions, and are easy and flexible to prepare by various physical and chemical approaches, and are therefore being developed and used for the degradation of organic pollutants and/or contaminants, splitting of water to generate hydrogen and oxygen, and conversion of solar energy to reduce carbon dioxide and produce chemical energy at room temperature or ambient temperature, which are important and interesting topics of scientific research over the past two decades [1,2,3,4,5,6]. Small enough optical bandgap semiconductors are being explored to allow efficient absorption overlap with the solar spectrum and to make effective use of solar energy, as visible light contains as much as 43% sunlight, for the development of visible-light-derived semiconducting photocatalysts [7,8].

Metal-free polymeric graphitic carbon nitride (g-C_3_N_4_), synthesized from earth-abundant elements of carbon and nitride, exhibits specific electrical properties due to special layer structures [9,10,11]. It has been widely used for photocatalytic water splitting to produce hydrogen and oxygen, as well as degradation of organic matter in visible light due to its superior optical properties and band structure feature (optical bandgap energy around 2.7 eV) [12,13]. The iron (Fe)-doped semiconducting photocatalytic material system is found to have enhanced electron transfer, leading to improved photocatalytic performance [11,14]. Bismuth molybdate (Bi_2_MoO_6_) is one of the most important members in Aurivllius oxide materials. The lattice structure of γ phase Bi_2_MoO_6_ consists of [Bi_2_O_2_]^2+^ layers sandwiched between [MoO_4_]^2−^ slabs, which favor electron conduction and absorb visible light. Bi_2_MoO_6_ has a narrower optical bandgap energy (2.5–2.8 eV) compared to typical wide-bandgap oxide photocatalysts such as TiO_2_ and ZnO (3.2–3.4 eV). It is a non-toxic inorganic material system and has excellent thermal and chemical stability, making Bi_2_MoO6 well suited as a reusable visible-light-driven photocatalyst [13,15,16].

However, both Fe-doped g-C_3_N_4_ and Bi_2_MoO_6_ exhibit rapid recombination of photogenerated electron–hole pairs, resulting in poor quantum yields, which limits and restricts their practical optoelectrical and optoelectrochemical applications [10,13,17]. Several approaches have been proposed to inhibit photoinduced carrier recombination, such as composition modification (i.e., impurity doping), textural microstructure optimization, and heterojunction structure design [18,19,20,21]. Bandgap engineering based on the heterojunction design of visible-light-driven photocatalysts is a promising and feasible route to increase the effective use of solar energy for high-speed chemical reactions and the treatment of organic pollutants in the environment [10]. The development of visible-light-derived photocatalysts based on coupling with graphitic carbon nitride (g-C_3_N_4_) and oxide semiconductor nanoparticles has attracted considerable attention and interest [12,13,17,18,19,22].

Both photocatalytic material systems, graphitic carbon nitride (g-C_3_N_4_) and metal oxide semiconductors, possessed simple fabrication procedures, cost-effectiveness, chemical inertness, thermal and long-term stability, non-toxic and environmentally friendly properties, and good visible light photocatalytic response. The development of visible-light-derived photocatalysts based on coupling with g-C_3_N_4_ nanostructures and oxide semiconductor nanoparticles has attracted considerable attention and interest. Recent studies have reported that composite photocatalysts of graphitic carbon nitride (g-C_3_N_4_) and bismuth-based oxide semiconductor materials (such as g-C_3_N_4_/Bi_2_O_3_, g-C_3_N_4_/BiVO_4_, g-C_3_N_4_/Bi_2_MoO_6_, and g-C_3_N_4_/Bi_2_WO_6_) to form type II or Z-scheme heterojunctions can enhance light adsorption capability and effectively inhibit or reduce the recombination of photogenerated electrons and holes, and then exhibit high photocatalytic activity.

In this study, two semiconductor materials with bandgap energies below 3.0 eV, Fe-doped g-C_3_N_4_ and Bi_2_MoO_6_, were selected as visible-light-derived photocatalysts and coupled to form Fe-g-C_3_N_4_/Bi_2_MoO_6_ composite to improve the photocatalytic activity and the photodegradation response performance of RhB in aqueous solution under visible light illumination at room temperature [23]. A comparative study of the structural characteristics, optical properties, and photocatalytic degradation efficiency between Fe-doped g-C_3_N_4_ particles, Bi_2_MoO_6_ particles, and Fe-g-C_3_N_4_/Bi_2_MoO_6_ composite was investigated and reported. In addition, a cyclic experiment was carried out to evaluate the repeatability and stability of the developed composite photocatalyst.

## 2. Results and Discussion

### 2.1. Physical Properties of Semiconducting Nano-Photocatalysts

**Figure 1:**
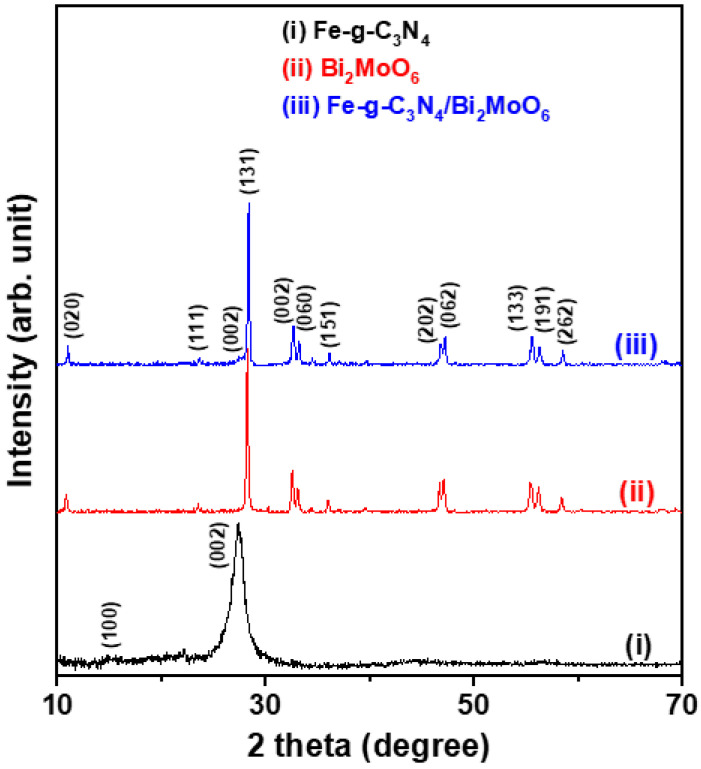
Powder X-ray diffraction (XRD) patterns of Fe-doped g-C_3_N_4_ particles, Bi_2_MoO_6_ particles, and Fe-g-C_3_N_4_/Bi_2_MoO_6_ composite.

Powder X-ray diffraction (XRD) patterns of Fe-doped g-C_3_N_4_, Bi_2_MoO_6_, and Fe-g-C_3_N_4_/Bi_2_MoO_6_ composite samples are shown in Figure 1. The two diffraction peaks of the Fe-doped g-C_3_N_4_ sample (pattern (i)) are indexed with the planes (100) and (002) and represent the typical diffraction pattern as pure g-C_3_N_4_ phase (JCPDS file No.87-1526) [11]. The twelve diffraction peaks, i.e., (020), (131), (002), (060), (062), (133) planes, matched well with the standard card of JCPDS 21-0102, confirming that the Bi_2_MoO_6_ sample has an orthorhombic crystal (patterns (ii) and (iii)) [18]. The average crystallite sizes of Fe-doped g-C_3_N_4_ and Bi_2_MoO_6_ samples were calculated from the main diffraction peaks for the (002) plane of Fe-doped g-C_3_N_4_ and the (131) plane of Bi_2_MoO_6_ using the Scherrer equation to be 4.3 nm and 38.6 nm (Table 1), respectively. In addition, the diffraction peaks of the Fe-g-C_3_N_4_/Bi_2_MoO_6_ composite agree well with the diffraction peaks of the Bi_2_MoO_6_ particles. T. Ma et al. presented similar XRD examination results [18]. Moreover, the average crystallite size of the Fe-g-C_3_N_4_/Bi_2_MoO_6_ composite (40.5 nm) is approximately 5% larger than that of the Bi_2_MoO_6_ particles (38.6 nm).

**Figure 2:**
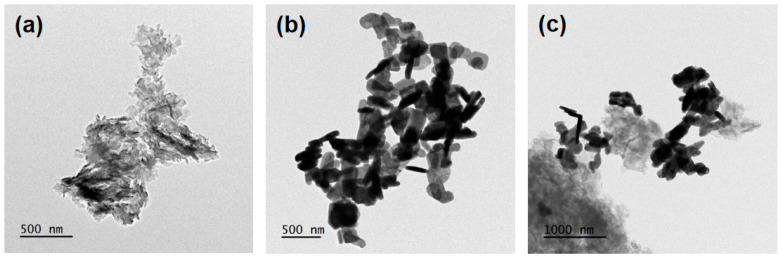
Transmission electron microscopy (TEM) micrographs of (**a**) Fe-doped g-C_3_N_4_ particles, (**b**) Bi_2_MoO_6_ particles, and (**c**) Fe-g-C_3_N_4_/Bi_2_MoO_6_ composite.

The morphology and particle size of the three obtained particle samples are characterized by TEM observation (Figure 2). ImageJ software version 2024 was used to estimate the particle size from each corresponding TEM image. These TEM micrographs show that the Fe-doped g-C_3_N_4_ sample exhibits an irregular and typical layered platelet morphology with an average particle size of 1860 nm (Figure 2a) and the Bi_2_MoO_6_ sample is composed of a roughly rectangular and rod-shaped granular morphology with an average particle size of 328 nm (Figure 2b). Figure 2c is a TEM micrograph of the Fe-g-C_3_N_4_/Bi_2_MoO_6_ composite showing a significant complex morphology with an average particle size of 1455 nm associated with Bi_2_MoO_6_ particles (dark region) and Fe-g-C_3_N_4_ (light grey region). It provides direct evidence to confirm the formation of heterojunction structures in the hybrid material. This feature can benefit from improving photoinduced charge separation and then preventing their rapid recommendation, as well as providing an efficient electron transfer route compared to pure Fe-doped g-C_3_N_4_ and Bi_2_MoO_6_ particles [10].

**Figure 3:**
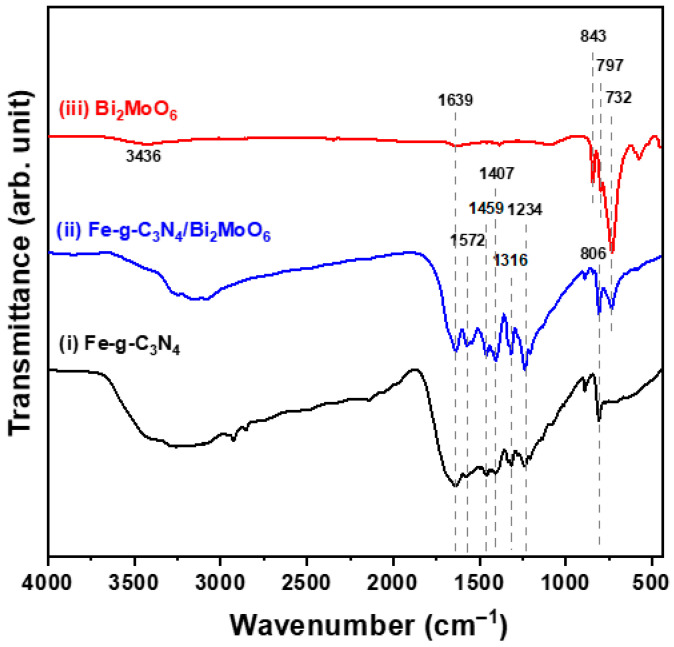
Fourier transform infrared (FTIR) spectra of Fe-doped g-C_3_N_4_ particles, Bi_2_MoO_6_ particles, and Fe-g-C_3_N_4_/Bi_2_MoO_6_ composite.

Measurement of the Fourier transform infrared (FTIR) spectrum is carried out to identify the functional groups and study the formation of compounds. The FTIR spectra of the three samples are shown in Figure 3. The absorption peak at 1639 cm^−1^ and the absorption band at about 3436 cm^−1^ are attributed to the stretching of the O-H bond and the deformation vibration of moisture absorption [24]. The FTIR spectra of the Fe-doped g-C_3_N_4_ particles (spectrum (i)) and the Fe-g-C_3_N_4_/Bi_2_MoO_6_ composite (spectrum (ii)) show little difference when the Bi_2_MoO_6_ and Fe-doped g-C_3_N_4_ particles are coupled. Two samples exhibit characteristic infrared absorption bands around 3000–3500 cm^−1^ and 1200–1700 cm^−1^, as well as a sharp absorption peak at 806 cm^−1^. The infrared absorption peak represents the typical respiratory mode of triazine units, which is specific for g-C_3_N_4_ [25,26]. According to previous reports, infrared absorption peaks in the 500–900 cm^−1^ range (spectrum (iii)) can be assigned to typical vibration modes of Bi_2_MoO_6_ lattices, including Bi-O and Mo-O bond stretching and Mo-O-Mo bridge stretching modes [18].

**Figure 4:**
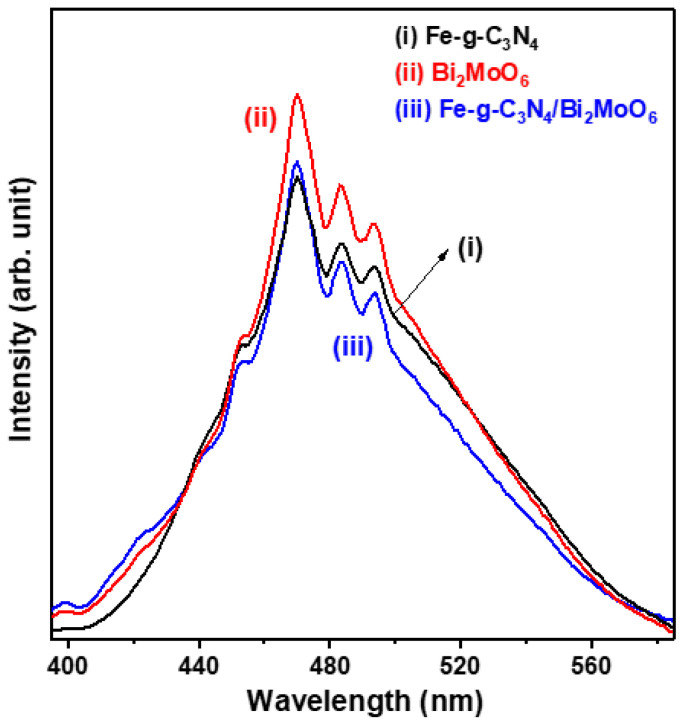
Room temperature fluorescence (FL) spectra of Fe-doped g-C_3_N_4_ particles, Bi_2_MoO_6_ particles, and Fe-g-C_3_N_4_/Bi_2_MoO_6_ composite.

The photoinduced charge transfer process of photogenerated electron–hole pairs was studied using the fluorescence (FL) spectrum. It is well known that the lower the intensity of the FL emission, the lower the recommended efficiency of the excited electrons and holes. Figure 4 shows room temperature FL spectra of Fe-doped g-C_3_N_4_ particles, Bi_2_MoO_6_ particles, and Fe-g-C_3_N_4_/Bi_2_MoO_6_ composite excited by 350 nm light. Three samples exhibit almost identical peak positions at 470, 483, and 493 nm. This suggests that they have a similar optical bandgap energy. Furthermore, the relative intensity of the peaks is of the order of Bi_2_MoO_6_ (spectrum (ii)) > Fe-doped g-C_3_N_4_ (spectrum (i)) > Fe-g-C_3_N_4_/Bi_2_MoO_6_ (spectrum (iii)). It should be noted that the recombination efficiency of photogenerated carriers for the Fe-g-C_3_N_4_/Bi_2_MoO_6_ composite is reliably lower than for Fe-doped g-C_3_N_4_ particles.

**Figure 5:**
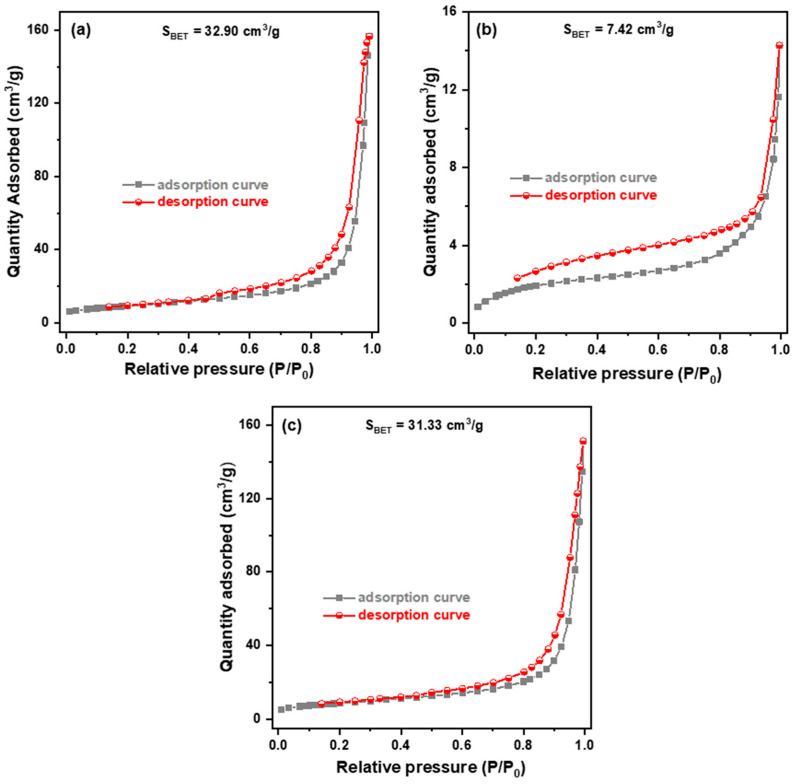
Nitrogen adsorption–desorption isotherms of (**a**) Fe-doped g-C_3_N_4_ particles, (**b**) Bi_2_MoO_6_ particles, and (**c**) Fe-g-C_3_N_4_/Bi_2_MoO_6_ composite.

Figure 5a–c show the low-temperature nitrogen adsorption/desorption isotherms for Fe-doped g-C_3_N_4_ particles, Bi_2_MoO_6_ particles, and Fe-doped g-C_3_N_4_/Bi_2_MoO_6_ composite, respectively. The BET surface areas of the three samples are summarized in the fourth column of Table 1. The Bi_2_MoO_6_ particles were found to have a smaller surface area (7.42 m^2^/g) than the Fe-g-C_3_N_4_ particles (32.90 m^2^/g). When Fe-g-C_3_N_4_ is coupled with Bi_2_MoO_6_ to form Fe-g-C_3_N_4_/Bi_2_MoO_6_, the surface area decreases slightly to 31.33 m^2^/g. The measured pore volume of three samples is also given in Table 1, which shows that its characteristic is the same as the surface characteristic.

**Figure 6:**
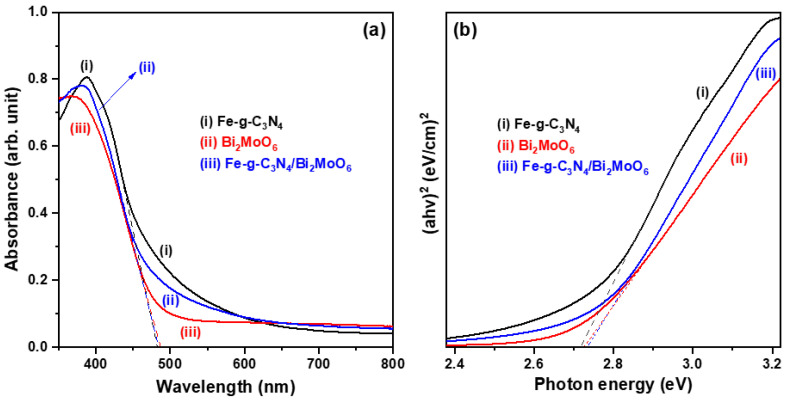
(**a**) Ultraviolet–visible diffuse reflectance spectroscopy (UV-Vis DRS) spectra and (**b**) Tauc plot of Fe-doped g-C_3_N_4_ particles, Bi_2_MoO_6_ particles, and Fe-g-C_3_N_4_/Bi_2_MoO_6_ composite.

The recorded UV-Vis DRS spectra of Fe-doped g-C_3_N_4_ particles, Bi_2_MoO_6_ particles, and Fe-g-C_3_N_4_/Bi_2_MoO_6_ composite are shown in Figure 6a. In the measured wavelength range, three samples exhibited a similar optical absorption spectrum. These spectra showed that the fundamental absorption edge of the Fe-doped g-C_3_N_4_ sample is at 483 nm and that the Bi_2_MoO_6_ sample has the absorption edge close to 485 nm. As the two different semiconductor particles are coupled, the Fe-g-C_3_N_4_/Bi_2_MoO_6_ composite sample has an absorption edge at 487.8 nm, showing a slight red shift. The optical bandgap energy of the prepared semiconductor samples can be calculated using the following Tauc relation [22,23]:

α(hv) = A (hv − E_g_)^1/2^, (1)

where hv is the photon energy, A is a constant, and α and E_g_ are the absorption coefficient and the optical bandgap energy (at wave vector k equal to zero) of the semiconductors. Tauc plots are based on the relationship between (αhv)^2^ and hv to determine the optical bandgap energy for direct transitions. By extrapolating the tangent lines of the drop part to the *x*-axis (photon energy, eV) in Figure 6b, the optical bandgap energies of Fe-doped g-C_3_N_4_, Bi_2_MoO_6_, and Fe-g-C_3_N_4_/Bi_2_MoO_6_ were determined to be 2.74 eV, 2.73 eV, and 2.72 eV, respectively. The edge potentials of the valence band (VB) and conduction band (CB) of a semiconductor at the zero charge point can theoretically be predicted by the following equations [10,27], which are related to the Mullikan electronegativity theory.

E_VB_ = χ − E_e_ − 0.5 E_g_, (2)

E_CB_ = E_VB_ − E_g_, (3)

where E_VB_ and E_CB_ are edge potentials of the valence band and conduction band, χ is the absolute electronegativity of the semiconductor (χ = 4.72 eV and 5.55 eV for Fe-g-C_3_N_4_ and Bi_2_MoO_6_), E_e_ is the free electron energy on the hydrogen scale, which is about 4.5 eV, and E_g_ is the optical bandgap energy of the semiconductor. Here, the VB and CB edge potentials for Fe-doped g-C_3_N_4_ and Bi_2_MoO_6_ are determined to be 1.58 eV, −1.14 eV and 2.41 eV, −0.32 eV, respectively. The flat band potentials of g-C_3_N_4_ and Bi_2_MoO_6_ semiconductors have been evaluated by the Mott–Schottky curves of S. We et al. and M. Xue et al. to be −1.19 eV and −0.32 eV, respectively [28,29]. Such characteristics are close to our calculated results.

### 2.2. Photocatalytic Degradation Performance of Semiconducting Nano-Photocatalysts

**Figure 7:**
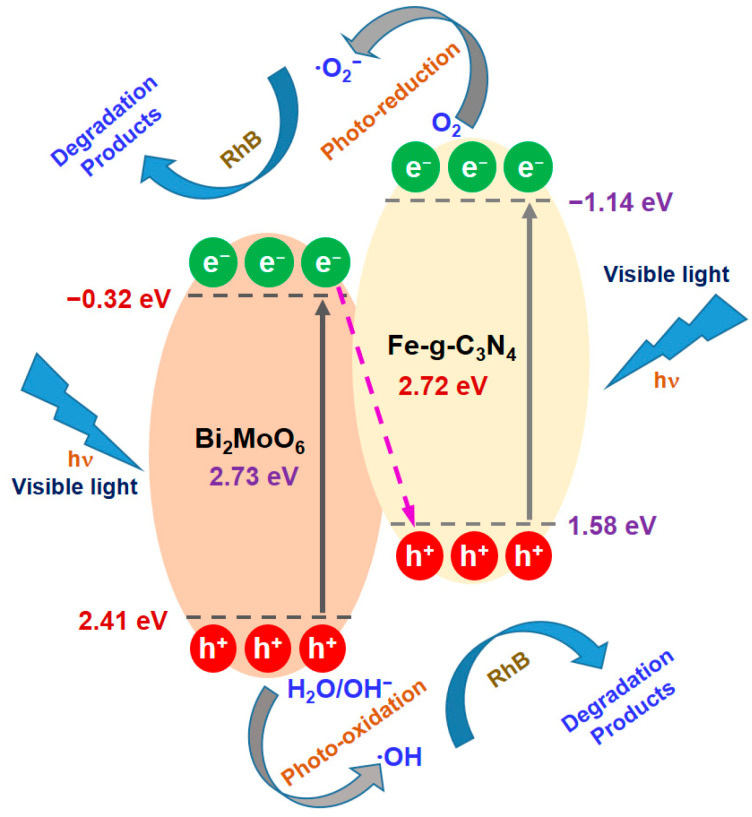
Schematic diagram of the possible photogenerated charge carrier, carrier transfer process, and photocatalytic degradation reaction mechanism of a heterostructured Fe-g-C_3_N_4_/Bi_2_MoO_6_ composite photocatalyst under visible irradiation.

Based on the analysis and discussion, a possible photocatalytic degradation mechanism of the Fe-g-C_3_N_4_/Bi_2_MoO_6_ composite is proposed, involving the photogenerated electron−hole pairs and photodegradation of organic pollutants. The heterojunction structure of Fe-doped g-C_3_N_4_ and Bi_2_MoO_6_ is shown in Figure 7, where both semiconducting materials are easily excited and generated electrons and holes after irradiation with visible light. Since both the CB and the VB edge potentials of Fe-doped g-C_3_N_4_ are more negative than those of Bi_2_MoO_6_, the photogenerated electrons in the CB of Fe-doped g-C_3_N_4_ could migrate to the CB of Bi_2_MoO_6_ and the photogenerated holes in the VB of Bi_2_MoO_6_ could migrate to the VB of Fe-g-C_3_N_4_. However, the electrons in the CB of Bi_2_MoO_6_ cannot reduce O_2_ to ^•^O_2_^−^ because its CB edge potential (−0.32 eV) is higher than the standard redox potential (E^0^ (O_2_/^•^O_2_^−^) = −0.046 eV vs. NHE). Similarly, the holes in the VB of Fe-doped g-C_3_N_4_ cannot react with H_2_O or OH^−^ near the surface of Fe-doped g-C_3_N_4_ to form ^•^OH (E^0^ (OH^−^/^•^OH) = 2.4 eV vs. NHE) due to its lower VB edge potential (1.58 eV).

As shown in the Fe-g-C_3_N_4_/Bi_2_MoO_6_ heterojunction material system of Figure 7, photogenerated electrons in the CB of Bi_2_MoO_6_ can quickly jump to the VB of Fe-doped g-C_3_N_4_ to combine the holes in Fe-doped g-C_3_N_4_, which feature could lead to the electron accumulation in the CB of Fe-doped g-C_3_N_4_ side and the holes in the VB of Bi_2_MoO_6_ side. Therefore, the electrons in the CB of Fe-doped g-C_3_N_4_ can capture adsorbed O_2_ on its surface to form ^•^O_2_^−^ and the holes in the VB of Bi_2_MoO_6_ could oxidize H_2_O or OH^−^ to form ^•^OH to achieve the photocatalytic degradation reaction [4]. That is the main physical mechanism for improving the photocatalytic performance of heterojunction-based composite photocatalysts.

The pH values of the single RhB aqueous solution, RhB aqueous solution with Fe-g-C_3_N_4_ nanoparticles, RhB aqueous solution with Bi_2_MoO_6_ nanoparticles, and RhB aqueous solution with Fe-g-C_3_N_4_/Bi_2_MoO_6_ nanocomposite were 5.08, 4.37, 4.93, and 4.50, respectively. We measured the pH value and monitored the visible transmission of mixed aqueous solutions of RhB dye and different developed photocatalysts and found that each mixed aqueous solution continued towards the neutral and clear with the photodegradation process. The photocatalytic degradation performance and activity of the semiconducting particles and the composite was evaluated by determining the photodegradation rate of the RhB aqueous solution under visible light irradiation.

**Figure 8:**
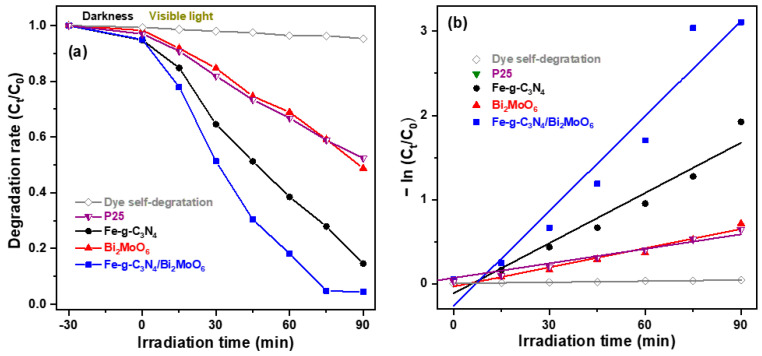
(**a**) Dynamic photocatalytic degradation and (**b**) linear transfer −ln (C_t_/C_0_) of the kinetic curves for degradation of the RhB aqueous solution at 10 ppm over three different semiconducting photocatalysts and Degussa P25 TiO_2_ nanoparticles under visible light irradiation.

Figure 8a shows the variation of the photocatalytic degradation rate (C_t_/C_0_, where C_0_ is the initial concentration of dye and C_t_ is the dye concentration after light irradiation time t) of RhB aqueous solution with visible light irradiation time for the three semiconducting photocatalysts [7]. The photodegradation efficiency of photolysis (i.e., without photocatalyst, only the RhB) by exposure to visible light for varying periods of time is negligible due to its relatively stable bonding structure. The dark adsorption capacity for 30 min of the Bi_2_MoO_6_ photocatalyst was 1.7%, and the those of the Fe-doped g-C_3_N_4_ and Fe-g-C_3_N_4_/Bi_2_MoO_6_ photocatalysts were about 5%. According to the measured results, the characteristic of the photodegradation efficiency with irradiation time for P25 TiO_2_ is similar to that of Bi_2_MoO_6_, and the RhB dye removal efficiencies of three types of photocatalysts were observed to follow the order Fe-g-C_3_N_4_/Bi_2_MoO_6_ > Fe-doped g-C_3_N_4_ > Bi_2_MoO_6_ under identical conditions. It is observed that the photocatalytic degradation efficiency of the Fe-g-C_3_N_4_/Bi_2_MoO_6_ composite photocatalyst can reach 95.20% after visible light irradiation for 75 min, and the degradation efficiencies for the P25 TiO_2_, Bi_2_MoO_6_, Fe-doped g-C_3_N_4_, and Fe-g-C_3_N_4_/Bi_2_MoO_6_ photocatalysts were 47.57%, 51.42%, 85.40%, and 95.53%, respectively, when under visible light irradiation for 90 min. Among the three semiconducting photocatalysts, the Fe-g-C_3_N_4_/Bi_2_MoO_6_ composite exhibits the highest photocatalytic performance towards the RhB aqueous solution because it has a large specific surface area and the heterojunction structure can effectively inhibit electron–hole recombination.

To quantitatively investigate the reaction kinetics of dye photodegradation, the degradation rate data were fitted by the pseudo-first-order approximation in a typical model of −ln (C_t_/C_0_) = k_a_t, where k_a_ is the apparent first-order rate constant (min^−1^) and t is the irradiation time [17]. As presented in Figure 8b, the apparent reaction rate constants for the P25 TiO_2_, Fe-doped g-C_3_N_4_, Bi_2_MoO_6_, and Fe-g-C_3_N_4_/Bi_2_MoO_6_ photocatalysts were 0.0058, 0.0199, 0.0076, and 0.0376, respectively (the last column of Table 1). It is worth noting that the reaction rate constant of Fe-g-C_3_N_4_/Bi_2_MoO_6_ photocatalyst is 4.95 times higher than that of the Bi_2_MoO_6_ photocatalyst and 1.9 times higher than that of the Fe-doped g-C_3_N_4_ photocatalyst. These results reveal that the photocatalyst has a large specific surface area, which provides more photodegradation reaction sites and leads to a high reaction rate. In addition, the interface between the heterojunction of two semiconductors plays a critical role in the photocatalytic activity.

**Figure 9:**
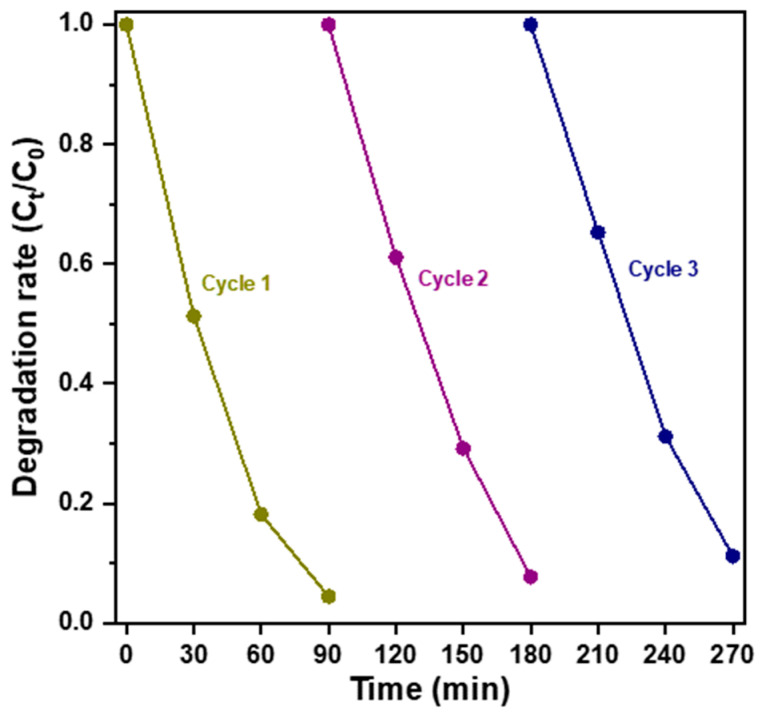
Comparison of the photocatalytic degradation performance of RhB aqueous solution at 10 ppm with three cycles for the Fe-g-C_3_N_4_/Bi_2_MoO_6_ heterostructured photocatalyst.

Since the Fe-g-C_3_N_4_/Bi_2_MoO_6_ composite photocatalyst exhibited the best photodegradation performance in the present study, we performed a cycling test to reuse it as the photocatalyst for treatment in a similar 10 ppm identical RhB aqueous solution under visible light for three cycles to explore stability and reproducibility. After three cycles, the degradation rate of the developed composite photocatalyst decreased to less than 7% (as shown in Figure 9), which may be due to loss of a small amount of photocatalyst during the cycling experiment. The cycling test showed that it was photostable and a good candidate for practical photoelectrochemical application.

## 3. Materials and Methods

### 3.1. Procedures for Preparing or Synthesizing Three Types of Visible Light Photocatalysts

The iron-doped graphitic carbon nitride (Fe-doped g-C_3_N_4_) was synthesized by the simple calcination method. A total of 4.0 g of melamine and 0.02 g iron(iii) nitrate nonahydrate [Fe(NO₃)₃·9 H₂O, Alfa Aesar] were dissolved in 20 mL of distilled water with 1 mL of hydrochloric acid (HCl, 37%, J.T. Baker) under magnetic stirring at 250 rpm for 30 min at room temperature. The resulting white suspension was dried at 80 °C for 8 h to remove the liquid phase and then heated at 2 °C/min up to 550 °C and held for 4 h in a box furnace without atmosphere protection to obtain the Fe-g-C_3_N_4_ product. A typical hydrothermal method was used to synthesize bismuth molybdate (Bi_2_MoO_6_) nanoparticles. Bismuth(III) nitrate pentahydrate (Bi(NO_3_)_3_·5H_2_O, Alfa Aesar) was dissolved in 2 mol/L nitric acid (HNO_3_, J.T. Baker) solution and sodium molybdenum oxide dihydrate (Na_2_MoO_4_·2H_2_O, Alfa Aesar) was also dissolved in 2 mol/L sodium hydroxide (NaOH, SHOWA) solution. The two resulting solutions were slowly mixed and stirred at 300 rpm for 30 min, then the pH of the mixed solution was adjusted to 5 to form a clear yellowish-white suspension. The prepared solution was transferred to a Teflon-lined stainless steel autoclave (Parr Instrument Company, model 4744, USA) and the synthesis of the hydrothermal reaction was maintained at 200 °C for 8 h. After natural cooling of the autoclave to room temperature, the Bi-based oxide precipitates were subjected to high-speed centrifugation, washed several times with distilled water and once with ethanol, and finally dried at 60 °C for 24 h to obtain the Bi_2_MoO_6_ product.

The heterojunction material coupled with Fe-doped g-C_3_N_4_ and Bi_2_MoO_6_ was prepared by a facile solution process by stirring and mixing the as-synthesized Fe-doped g-C_3_N_4_ nanostructures and the Bi_2_MoO_6_ nanoparticles (weight ratio is 3:1) in ethanol at 400 rpm for three days at RT and then drying at 60 for 24 h and grinding to form the composite photocatalysts [30]. All chemicals used in this study were analytical-grade reagents.

### 3.2. Characterization of Physical Properties and Measurement of Photocatalytic Performance

The phase structure and crystallinity of these as-prepared compound particles were examined using a Bruker D8 Discover X-ray diffractometer (Bruker, Billerica, MA, USA) with Cu Kα radiation in the 2θ scanning range from 10° to 70°. Morphology observation and particle size estimation of three types of particle samples were investigated using a JEOL JEM2100F transmission electron microscope (TEM, Tokyo, Japan). Infrared absorption spectra were recorded using a PerkinElmer Frontier Fourier Transform Infrared spectroscopy (FT-IR, Waltham, MA, USA) in the frequency range 400 to 4000 cm^−1^. Fluorescence emission spectra were recorded at room temperature using a Shimadzu RF-5301PC spectrofluorophotometer (Kyoto, Japan) with a xenon lamp as the 350 nm excitation light source. The near UV-Vis absorption spectrum of each sample was recorded on a JASCO V-770 UV-Vis/NIR spectrophotometer (Oklahoma, OK, USA), which was used as a standard UV-Vis diffuse reflectance experiment in the wavelength range of 300−800 nm. The specific surface area value for each prepared particle sample was measured using nitrogen adsorption/desorption isotherms on a Micromeritics ASAP2020 surface area and porosimetry analyzer (Norcross, GA, USA) using the Brunauer–Emmett–Teller (BET) method.

The photocatalytic activities of three as-prepared semiconducting photocatalysts were evaluated by photodegrading the aqueous solution of Rhodamine B (RhB) under visible light irradiation for different times (from 0 to 90 min at 15 min intervals) using a xenon lamp, with a maximum power of 500 W, and a cut-off filter (λ ≥ 400 nm) as light source. The distance between the light source and the Pyrex glass cell was about 60 cm. To investigate the photodegradation efficiency, 100 mg of each photocatalyst was dispersed in an aqueous solution of RhB dye (100 mL, 10 mg/L). Before irradiation for the degradation reaction, each photocatalyst suspension should be kept in the dark for 30 min to ensure that there is sufficient contact and adsorption/desorption equilibrium between the photocatalyst and the organic dye. The mixed solution was then exposed to a fixed power of 300 W of visible light for the desired time. Before and after exposure to visible light, the concentration of the RhB aqueous solution was determined by measuring the absorbance characteristic on a Hitachi U-2900 spectrophotometer (Tokyo, Japan).

In order to identify the influence of semiconductor photocatalysts on degradation reaction activity ability for RhB dye, a blank experiment without photocatalysts was performed as a photolysis. We also selected Degussa (Evonik) P25 titanium dioxide (TiO_2_) nanoparticles as a reference sample to compare the photodegradation reaction rate and efficiency with the three developed semiconductor photocatalysts. To evaluate the recyclability and stability of the composite photocatalysts, cycling test was carried out. To do this, the suspension was collected for the next measurement by centrifugation at 500 rpm for 5 min at set time intervals.

## 4. Conclusions

This work provides a feasible and effective route for the preparation of visible-light-driven heterojunction photocatalyst based on Fe-g-C_3_N_4_ particles and Bi_2_MoO_6_ particles and demonstrated the potential for excellent degradation capacity of organic pollutants in wastewater. The heterostructured Fe-g-C_3_N_4_/Bi_2_MoO_6_ composite photocatalyst exhibited better photocatalytic performance, and higher photocatalytic activity than those of single Fe-g-C_3_N_4_ and Bi_2_MoO_6_ semiconductor photocatalysts was achieved for the photodegradation of RhB aqueous solution under visible light irradiation. The cycling test demonstrated that the developed composite photocatalyst is reproducible and stable for photoelectrochemical application.

## Figures and Tables

**Table 1:** Comparison of microstructural characteristics, optical bandgap, and photocatalytic activity of Fe-doped g-C_3_N_4_ particles, Bi_2_MoO_6_ particles, and Fe-g-C_3_N_4_/Bi_2_MoO_6_ composite.

Photocatalyst	Average Crystallite Size (nm)	Average Particle Size (nm)	S_BET_ (m^2^/g)	Pore Volume (cm^3^/g)	Optical Bandgap (eV)	Photodegradation Efficiency (%)	Reaction Rate Constant (min^−1^)
Fe-g-C_3_N_4_	4.3	1860	32.90	0.243	2.74	85.40	0.0199
Bi_2_MoO_6_	38.6	328	7.42	0.013	2.73	51.21	0.0076
Fe-g-C_3_N_4_/Bi_2_MoO_6_	40.5	1455	31.33	0.234	2.72	95.53	0.0376

## Data Availability

Experimental results and data are presented in this article.
